# Communications Between Peripheral and the Brain-Resident Immune System in Neuronal Regeneration After Stroke

**DOI:** 10.3389/fimmu.2020.01931

**Published:** 2020-09-18

**Authors:** Fangxi Liu, Xi Cheng, Shanshan Zhong, Chang Liu, Jukka Jolkkonen, Xiuchun Zhang, Yifan Liang, Zhouyang Liu, Chuansheng Zhao

**Affiliations:** ^1^Neurology, The First Hospital of China Medical University, Shenyang, China; ^2^A.I. Virtanen Institute and Institute of Clinical Medicine/Neurology, University of Eastern Finland, Kuopio, Finland; ^3^Stroke Center, The First Hospital of China Medical University, Shenyang, China

**Keywords:** brain, peripheral, immune system, gut microbiota, communication, stroke, neuronal regeneration

## Abstract

Cerebral ischemia may cause irreversible neural network damage and result in functional deficits. Targeting neuronal repair after stroke potentiates the formation of new connections, which can be translated into a better functional outcome. Innate and adaptive immune responses in the brain and the periphery triggered by ischemic damage participate in regulating neural repair after a stroke. Immune cells in the blood circulation and gut lymphatic tissues that have been shaped by immune components including gut microbiota and metabolites can infiltrate the ischemic brain and, once there, influence neuronal regeneration either directly or by modulating the properties of brain-resident immune cells. Immune-related signalings and metabolites from the gut microbiota can also directly alter the phenotypes of resident immune cells to promote neuronal regeneration. In this review, we discuss several potential mechanisms through which peripheral and brain-resident immune components can cooperate to promote first the resolution of neuroinflammation and subsequently to improved neural regeneration and a better functional recovery. We propose that new insights into discovery of regulators targeting pro-regenerative process in this complex neuro-immune network may lead to novel strategies for neuronal regeneration.

## Introduction

Stroke is one of the leading causes of functional deficits and death for aged populations. The lack of blood flow in ischemic conditions causes irreversible damage to neurons, and this disrupts the functioning of many neuronal networks. As a result, more and more attention has been paid in searching for therapies that can promote neuroplasticity. SVZ (subventricular zone) and SGZ (subgranular zone) are important areas for the proliferation of NPCs (neural precursor cells). The robustly increased proliferation of NPCs in these areas serves as the first step of neurogenesis after an ischemic stroke, and then newly generated neuroblasts migrate to the peri-infarct area, differentiate into mature neurons, and finally are able to compensate for the damaged neural network (1). Axonal regeneration is another critical component of the post-stroke neuronal regeneration process. This refers to certain conditions such as budding, growth and extension, and re-contacting of axons with their target cells to re-establish neural control and restore functions. These newly generated connections provide an anatomical basis for compensating for the loss of function after stroke. Therefore, both positive and negative regulators of neuronal regeneration need to be manipulated if one hopes to achieve a better functional outcome.

**Figure 1:**
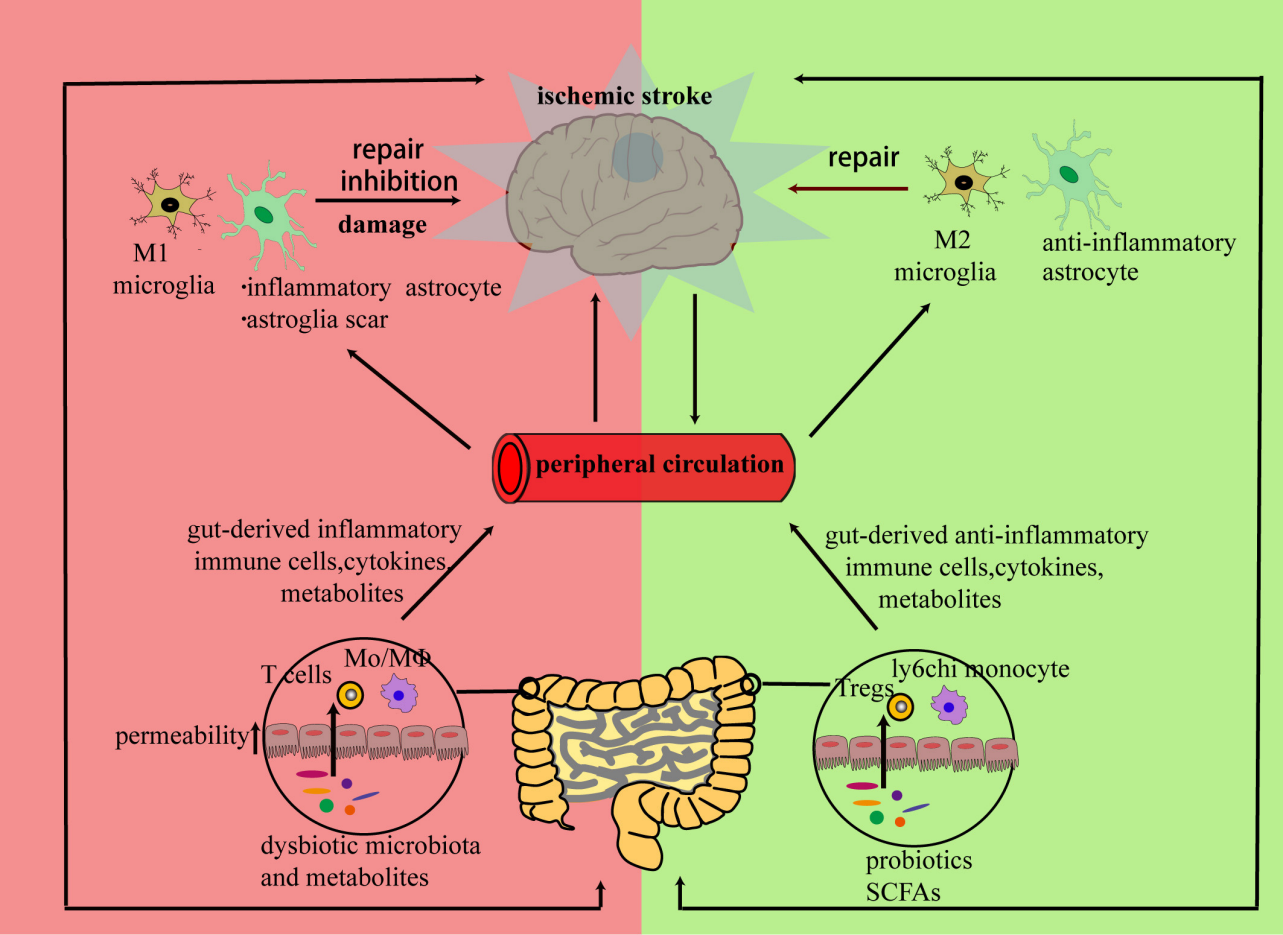
Brain and peripheral immune responses cooperate to repair a neural network damaged by ischemia: the inflammatory response triggered by neural debris may cause alterations in both brain and peripheral immune components. In the subacute or chronic stage of stroke, peripheral immune cells, cytokines, or microbiota metabolites from bone marrow, blood circulation, or gut can infiltrate the stroke-damaged brain and promote a bi-directional communication between resident and infiltrated cells. Then, the infiltrated cells can either exert direct regulatory effects on neurons or promote regulatory effects on the polarization of resident immune cells to initiate neuronal repair.

Previous studies have mainly focused on promoting neuroplasticity through growth factors and neurotrophic factors. These growth factors and neurotrophic factors also have immunomodulatory effects. Neurotrophic factors from NGF (nerve growth factor), GDNF (glial cell-derived neurotrophic factor), and BDNF (brain-derived neurotrophic factor) may attenuate the neurotoxic inflammatory response after a stroke (2). The brain also has its own resident immune cells such as microglia. Microglia together with infiltrated immune components initiate the immune response in the ischemic brain. However, changes in immune components are not only present in brain. Brain antigens can also be captured by peripheral immune cells and shape peripheral immune environment (3). This long-lasting immunological communication between brain and the periphery has been studied to mitigate ischemic injury in previous studies and also provides a new target for strengthening the repair process during the late stage of stroke (4, 5). As a result, either strengthening the repair-promoting immune responses or weakening the neurotoxic immune responses may promote neuroplasticity after stroke. Gut microbiota and their metabolites are also essential components of this repair system. As well as having a regulatory effect on the gastrointestinal tract, altered gut microbiota can also regulate brain function through neural, metabolic, endocrine, and immune mechanisms via the brain–gut axis (6). Recent studies have revealed that there are changes in gut microbiota related with the brain damage caused by a stroke and, furthermore, that these can influence the functional outcome after a stroke (7). The mechanisms by which gut microbiota influence functional recovery after a stroke are not fully clarified; their pivotal roles in bi-directional neuro-immune crosstalk are thought to regulate the integration and function of neural networks. Here, we first review some of the evidence emerging from experiments examining how communications between the peripheral immune response and neuroinflammation after stroke might be beneficial for neuronal regeneration and the functional recovery after a stroke. Then, the roles of gut microbiota in this immunological crosstalk are discussed. Finally, the potential benefits of immunomodulatory therapy will also be evaluated in this review. An overview of the bi-directional neuro-immune communications occurs in neuronal repair after stroke is schematized in Figure 1.

## Peripheral Immune Components Influence Neuronal Repair After Stroke

### Infiltrating Peripheral Immune Cells and Their Functions After Stroke

There is evidence pointing to the existence of communications between immune components in brain and peripheral circulation, and thus, it may be possible to target these systemic immune responses as a way of influencing the stroke outcome. Following cerebral ischemia and reperfusion damage, resting microglia are activated and release chemo-attractive cytokines; this allows the infiltration of peripheral myeloid cells through the disrupted blood–brain barrier and initiates the innate stage of immune response (8). After the innate stage, the release of brain antigens causes dendritic cell (DC) precursors to infiltrate quickly into the brain; these cells exist in the brain for a long time and it has been claimed that they determine stroke outcome by influencing the long-term T cell response (9). Brain antigens can also be captured by antigen-presenting cells in cervical lymph nodes; these are subsequently presented to different types of T cells in the cervical lymph nodes to initiate an adaptive immune response (3). In this section, we will review the infiltration of immune cells and their functions in detail.

#### Monocytes and Macrophages

In the acute phase of stroke, bone marrow-derived peripheral monocytes are recruited into the brain through a CCR2 (chemokine receptor 2)-dependent mechanism and their numbers peak at day 3 to day 4 after stroke (10). The two subtypes of Mo/MΦ (monocyte/macrophage) have different spatial distributions: CCR2^+^ Mo/MΦ are mainly located in the ischemic core and can be switched into alternatively active CX3CR1^+^ Mo/MΦ in the subacute and chronic phase, while CX3CR1^+^ Mo/MΦ mainly surround the ischemic area (11). The long-term existence of infiltrated Mo/MΦ suggests that they can also participate in neural repair after stroke. Infiltrated Mo/MΦ first upregulate wound-healing genes and then shift to pro-inflammatory ones in the ischemic microenvironment, leading to neurotoxic inflammatory injury and compromised endogenous neurogenesis (12, 13). However, they gradually switch into an anti-inflammatory and pro-regenerative phenotype and contribute to the neural repair process in chronic stage (14). These cells cooperate with resident microglia and macrophage to promote neuroinflammation resolution through efferocytosis of neural debris, and then they show a genomic reprogram and upregulate genes related with neurovascular plasticity biological processes (15, 16). CCL2/CCR2 signaling, which mediates the invasion of monocytes, has been demonstrated to promote neural differentiation and neurite elongation (17). The functions of Mo/MΦ have been further demonstrated by the fact that after CCR2 antagonist treatment, there is a suppression of neural repair and a worse outcome (18). Mo/MΦ also cooperate with other immune cells such as regulatory T cells to potentiate neuro-regeneration. Monocyte-derived macrophages regulate the recruitment of regulatory T cells into damaged CNS tissues, which is also beneficial for the repair process after stroke (19). Collectively, infiltrated Mo/MΦ functions alone or through interaction, show a beneficial effect in the post-stroke neural repair process. Future studies can be conducted from two perspectives: The first is identifying the precise time course of pro-regenerative Mo/MΦ activation. The second is finding extracellular and intracellular signalings that control the pro-regenerative conversion of infiltrated Mo/MΦ.

#### Regulatory T Cells

In stroke models, it has been demonstrated that immune components in both the brain and periphery can communicate and contribute to the expansion of Treg cells, leading to a long-term immunomodulatory effect after stroke (20). The massive accumulation and infiltration of lymphocytes can persist in ischemic brain even in the delayed phase after a stroke (4). Anti-inflammatory cytokines from regulatory T cells such as IL-10 and TGF-β interact with both positive and negative regulators of neural regeneration and influence the repair process after a stroke. After binding to its receptor, the classic anti-inflammatory cytokine, IL-10, protects neurons from apoptosis after ischemic injury, promotes neural stem cell proliferation in SVZ, and promotes neural progenitor differentiation and neurite outgrowth through phosphorylating PI3K/AKT and JAK/STAT-3 (21–23). IL-10 also neutralizes the properties of pro-inflammatory cytokines and increases the levels of nerve growth factors to preserve neural integration by the inhibition of caspase-3 activity (24). Therefore, a specific depletion of regulatory T cells can lead to a reduction in the number of NPCs (25). TGF-β is another anti-inflammatory cytokine with growth-promoting properties. The anti-inflammatory effect of TGF-β may limit neuroinflammation during the subacute phase after a stroke. GDF10 (growth and differentiation factor 10) is a member of the TGFβ superfamily; its levels are upregulated in the peri-infarct area during the initiation of axonal sprouting (26). GDF10 downregulates PTEN, upregulates PI3K signaling, and induces axonal guidance factors to promote axonal sprouting after ischemic damage (26). However, another key member of the TGFβ superfamily, BMPs (bone morphogenetic proteins), exerts an inhibitory effect on brain repair. The increased expression of BMPs can be detected from 1 week after the stroke and lasts for 4 weeks; these agents participate in the repair process by modulating astrogliosis (27). Treatment with noggin, an endogenous antagonist of BMP, was able to attenuate glial scar formation, increase axon growth-related protein GAP-43, and elevate the number of M2 microglia present in ipsilateral ischemic brain (28), which are all beneficial with respect to axonal repair. Noggin also shifted reactive microglia to an iron-releasing state and promoted remyelination in the ischemic brain (29). In addition to the anti-inflammatory cytokines, other products from Treg cells are also beneficial for neural network reconstruction after stroke. CCN3 (Cellular Communication Network Factor 3) from Treg cells has been confirmed to contribute to the axonal and myelin repair process following an ischemic injury (30). Hence, regulatory T cells and their products may provide new perspectives for neuronal regeneration after stroke.

#### Cytokines From Infiltrated Immune Cells

After a stroke, DAMPs (damage-associated molecular patterns) released from cellular debris cause IL-23 secretion from microglia and macrophage via TLRs (Toll-like receptors), and then brain-resident microglia, infiltrating Th17 cells, and γδ T cells activated by IL-23 can release the neurotoxic inflammatory cytokines (31). NF-κB and CREB are essential transcription factors in inflammatory cascades; inflammatory factors like IL-17, IL-1, and IFNγ may shift the balance between NF-κB and CREB toward NF-κB domination until the chronic phase of stroke, which may cause neural damage and inhibit neural plasticity (32). After binding to endothelial IL-1R1 (interleukin 1 receptor, type I), IL-1 triggers the release of pro-inflammatory factors from activated microglia, promotes the invasion of leukocytes, and exerts inhibitory effects on neurogenesis (33). Pro-inflammatory IFNγ can cause neurodegeneration and mediate the detrimental effects of T cells on neurogenesis in the SVZ area (34). Some existing evidence support the proposal that inhibiting NF-κB-mediated inflammatory cascades seems to be a promising approach to potentiate post-stroke neural repair process. Elevated CREB concentration promotes axonal circuit plasticity and improves functional recovery after a stroke (35). The potential mechanism can be attributed to the downregulation of inhibitors of axonal regeneration including MAG (myelin-associated glycoprotein), Nogo, and oligodendrocyte myelin glycoprotein, and induction of neural repair-related FGF (fibroblast growth factors), GDNF, and their receptors (35, 36). Stimulating axon outgrowth and neurogenesis-related PI3K signaling also upregulates CREB and downregulates NF-κB through GSK-3β inhibition, which also polarizes microglia so that they adopt a repair-related M2 subtype (37). In addition to NF-κB, inflammatory cytokines can also inhibit axonal growth through the inhibition of TGF-β-induced Smad2/3 and p38 MAPK signaling (38).

However, there is evidence to suggest pro-inflammatory cytokines in contrast to their detrimental effects may also be a positive regulator of neuroplasticity. Elevated levels of IL-6 and TNF-α in the spinal cord after stroke upregulate the concentrations of GAP-43 (growth-associated protein 43), NT-3 (neurotrophin-3), and BDNF, all of which are known to be involved in spinal axonal plasticity and lead to better spontaneous post-stroke recovery (39). After neutralizing IL-6 with a specific antibody, the proliferation and differentiation of neural stem cells in SVZ was found to be significantly attenuated (40). A recent study about brain repair after traumatic brain injury suggests that repopulated microglia may serve as an important source of IL-6 and initiate pro-regenerative IL-6 trans-signaling, and then the IL-6/sIL-6R complex in neurons activated gp130 and downstream STAT3 pathway to promote the repair of injured brain (41). All these suggest that stroke-induced neuroinflammation is a double-edged sword in neural functional recovery, and it should be precisely manipulated to create a favorable environment for neuro-regeneration.

### Gut Microbiota as Potential Regulators of Neural Repair

More and more studies have shown that gut microbiota and their metabolites not only are an important part of the peripheral immune system but also exert a regulatory effect on the bi-directional gut–brain axis. Central nervous system diseases including stroke may cause a secondary dysfunction of gastrointestinal tract and alter the composition of the gut microbiota (42). A reduced diversity of microbiota species and an overgrowth of bacteria are the main features of post-stroke microbiota dysbiosis, which polarizes immune cells both in gut and brain to adopt a pro-inflammatory phenotype, and this may influence the stroke outcome (43). The leaky gut barrier caused by a stroke also leads to the translocation microbiota and metabolites to trigger neuroinflammation and a peripheral immune response (44). Accumulating evidence suggest that altering the gut microbiota composition can shape the local immune environment in the brain in favor of neurogenesis and axon growth after stroke because lymphocytes have been shown to migrate from gut to brain after stroke: Gut-derived CD4^+^ T cells migrate to meninges and control the balance between M1 and M2 microglia/macrophage after ischemic injury (45). Transplantation of dysbiotic microbiota into germ-free mice was claimed to shift T cells in gut and brain so that they adopted a pro-inflammatory polarization and enlarged the area of infarct (43). In contrast, treatment with gut-derived *Prevotella histicola* suppressed neuroinflammation; the mechanism was mediated by a regulation of systemic immune responses due to an increase in anti-inflammatory cells like Treg cells (46). The PSA (polysaccharide A) present on *Bacteroides fragilis* may stimulate the migration of CD103 expressing DCs to cervical lymph nodes and may inhibit demyelination by promoting the conversion of native CD4^+^ T cells to Treg cells (47). In addition to T lymphocytes, macrophage and monocytes are also involved in the repair-promoting effect of gut microbiota. Mice treated with antibiotics or CCR2 antibody experience a decrease in neurogenesis due to the lower number of ly6c^hi^ monocytes (48).

A different form of immune-modulatory effect in the microbiota–gut–brain crosstalk can be exerted by the metabolites of gut microbiota, such as SCFAs (short-chain fatty acids). Gut microbiota are important potential mediators of adult neurogenesis after stroke, as evidenced by SCFA-enriched fecal microbiota transplantation or butyric acid supplement, which have both been able to promote neurological recovery (49). SCFAs can modulate immune homeostasis not only in gut but also in the peripheral immune system and brain; both immune cells from peripheral circulation and those resident in the brain are targets of SCFAs (50). SCFAs generated by microbiota can induce the production of IL-10 from differentiated Th1 cells in gut and contribute to homeostasis in gut through STAT3 and mTOR signaling (51). Butyrate and propionate together enable DCs to promote extrathymic generation of Treg cells (52). After binding to Ffar2 (GPR43) on T cells, SCFAs inhibit the function of HDAC to promote the expression of transcription factor Foxp3, which leads to elevated numbers of colonic Treg cells (53). Activation of another butyrate receptor, Gpr109a, promotes the formation of anti-inflammatory colonic macrophages and DCs and this enables the induction of Treg cells (54). T cells activated by SCFAs then regulates the function of brain-resident microglia to promote synaptic plasticity after a stroke (55). It could be speculated that it is the migration of lymphocyte from gut to brain that mediates the modulatory effects of SCFA on neuroinflammation, as confirmed in a recent study: Transferring Treg cells from SCFA pre-treated mice reduced autoimmunity and inhibited axonal damage in the recipient mice, the authors speculated that the MAP kinase family and lipin-2 may have mediated the resolution of neuroinflammation (56). In summary, gut microbiota functions as a potential regulator of the brain's infiltrated peripheral immune cells and may, in this way, contribute to the repair process occurring after stroke. Further researches can be carried out to find out which microbiomes influence neural repair process and then verify their roles through fecal microbiota transplantation.

## Alterations in Brain-Resident Immune Cells Can Promote Neuronal Repair After a Stroke

### Resident Immune Cells and Neuroplasticity

Microglia are the brain-resident macrophages that function as the first immune responder and are also important contributors to the repair of damaged neural networks in ischemic brain. A recent study has demonstrated that efficient regeneration of damaged axons involves necroptosis of pro-inflammatory microglia followed by a repopulation of anti-inflammatory and pro-regenerative microglia (57). The cells may shift their polarization; i.e., they can exist either as the proinflammatory M1 type or the anti-inflammatory M2 type depending on the type of stimulation such as that present in cerebral ischemia and inflammation. The M2 type can be further divided into M2a, M2b, and M2c subtypes. M2a and M2c microglia exert anti-inflammatory and reparative properties, while M2b microglia mainly produce an anti-inflammatory cytokine (58). Thus, shifting the polarization of microglia seems to be a promising method to promote neuroplasticity after stroke. Clearance of dead cell debris by microglia serves as the first step in the repair process, since this prevents secondary inflammation and creates a favorable environment for neural repair and functional recovery after stroke. Soluble CX3CL1 released from dead neurons can bind to CX3CR1 on microglia and then trigger the phagocytic process to clear not only debris but also the unfavorable factors preventing neural repair (59). Then, alternatively activated microglia in SVZ serve as a source of IGF-1 (insulin-like growth factor 1) and also directly contribute to neural stem cells proliferation, differentiation, and migration to the striatum (60). Injured neurons can also release LCN2 (Lipocalin-2), which can induce microglia to polarize into the repair associated form, increase the release of anti-inflammatory cytokines and synaptic proteins, and exert neuroprotective effects (61). However, there is a less beneficial side to activated microglia as they have detrimental effects on neuroplasticity by producing IL-1β, which can trigger p53-mediated neural cell death (62). Therefore, promoting microglia M2 polarization or inhibiting M1 polarization seems to be a feasible method for neuronal repair after stroke.

Although astrocytes are not considered as typical brain-resident immune cells, they also show regulatory functions in both the innate and adaptive immune response (63). Following ischemic injury, astrocytes become activated and contribute to endogenous neural repair. Through secreting neuroblast attractive SDF-1 (stromal cell-derived factor-1), astrocytes contribute to the migration of neuroblasts to infarct area and compensate for the neural death caused by stroke (64). Furthermore, astrocytes can be reprogrammed into a neurogenic state and then be converted to neurons when Notch signaling is downregulated (65). In addition to regulating neurogenesis, astrogliosis also plays a controversial role in axon extension. On one hand, activation of neurotoxic A1 reactive astrocytes and inhibitory molecules from glia scar restrict axonal repair (66, 67). On the other hand, the glia scar serves as a physical and molecular wall surrounding the damaged area in an attempt to localize the ischemic lesion (68) and also release trophic factors (64), thus creating favorable milieus for axon regeneration. So, the formation of glial scar needs to be differentially manipulated at different stages of recovery rather than merely inhibiting scar formation in order to promote axonal extension.

### Interactions Between Microglia and Infiltrated Cells

Following ischemic injury, immune system, and related signaling may interact to promote the activation and polarization of microglia, and this process plays a crucial role in post-stroke neural repair. The intense pro-inflammatory stimulus in the microenvironment after a stroke activates microglia and upregulates M1 microglial TREM1 expression through NF-κB (69). Increased numbers of M1 microglia may further function as a source of inflammatory cytokines and facilitate the infiltration of peripheral immune cells. The M2 polarization of microglia can also be shaped by infiltrated immune cells. Chemokines and their receptors represent a target for communications between infiltrated monocyte/macrophage and resident cells, so they serve as a promising therapy for neural repair after stroke. Infiltrating Ly6C(hi) monocytes with CCR2 can promote M2 macrophage polarization (70), and this may induce a similar axonal repair process in a spinal cord injury (71). Another member of Chemokine Receptor family, CCR5, is commonly expressed on brain-resident cells like neurons, microglia, and infiltrated Mo/MΦ, which can be upregulated after an ischemic stroke (72). Several studies have indicated that maraviroc, a CCR5 antagonist can inhibit excessive pro-inflammatory responses caused by imbalanced CNS-peripheral immune crosstalk. In a spinal cord injury model, CCR5 blockade stimulated the release of an anti-inflammatory cytokine, which may further activate PPAR γ signaling and then shift the polarization of microglia toward the repair favoring M2 type (73). Treatment with the CCR5 inhibitor maraviroc in experimental autoimmune encephalitis (EAE) mice has also been reported to resolve neuroinflammation by attenuating inflammatory T cell infiltration without affecting anti-inflammatory regulatory T cells (74). More importantly, a recent study confirms that CCR5 inhibition after a stroke promotes neuroplasticity. Administration of AAV-shCCR5 or pharmacological knockdown of CCR5 with maraviroc leads to enhanced axonal sprouting to the contralateral motor cortex, possibly through an upregulation of CREB and DLK signaling in neurons, a downregulation of astrocyte reactivity, and a lack of inflammatory macrophage recruitment (72). In addition to chemokines and receptor signaling, the release of anti-inflammatory cytokines from infiltrated and resident cells is also important for the M2 polarization of microglia. TGF-β1 produced in the ischemic core may diffuse and upregulate TREM2 (triggering receptor expressed on myeloid cells 2) expression on NG2 (Neuron-glial antigen 2) chondroitin sulfate proteoglycan positive microglia in the peri-infarct area, which may promote phagocytosis of neural debris (75). After clearance of the debris, the main components of adaptive immunity T cells are often activated and the crosstalk between T cells and microglia may create a favorable immune environment for neural regeneration. Regulatory T cells can restrain LPS-induced inflammatory response of microglia via IL-10 secretion, and astrocyte-Treg interaction also contributes to this anti-inflammatory effect (76). In the experimental stroke model, exogenous delivery of the neuroprotective T cell cytokine, IL-33, also promotes anti-inflammatory IL-10 and IL-4 secretion, which may limit the extent of the neuroinflammation, reduce the infarct area, shift microglia toward the M2 polarization, and promote neural repair (77, 78). Using evidence learned from the LPS-induced neuro-inflammation model, we propose that Treg cells are mediators of IL-33's anti-inflammatory functions on microglia. Cyclic AMP signaling has long been considered to be related with neurogenesis and axon growth; e.g., when combined with Th2 cytokines, cAMP signaling can promote M2 polarization more efficiently (79). Since there exists a bi-directional crosstalk between infiltrated immune cells and resident microglia, microglia also show a regulatory effect on the infiltration of peripheral immune cells. M2 polarized microglia are important contributors to the release of anti-inflammatory cytokines following ischemic injury; these cytokines may further increase the proportion of anti-inflammatory immune cells and be beneficial for neural regeneration (80). This suggests that microglia polarization serves as a potential target for elevating anti-inflammatory and repair-promoting interactions between microglia and infiltrated peripheral cells. The therapeutic potential of promoting microglia M2 polarization by Fasudil supplement has been explored in the EAE model, in which the downregulation of IL-17 secretion from T cells was mediated by M2 polarized microglia after Fasudil treatment (81). Collectively, interactions between microglia and peripheral immune cells show a repair-promoting effect mainly through promoting subtype conversion of microglia. Future exploratory studies can be conducted to find mechanisms under this subtype conversion and then develop potential therapeutic methods targeting this immune crosstalk.

### Interactions Between Astrocytes and Infiltrated Cells

Astrocytes can both respond to and regulate neuroinflammation. MAPK, NF-κB, and the STAT3 pathways are considered shared mediators of the astrocytic immune response (63). Accumulating evidence suggest that astrocytic immune response shows a regulatory effect on post-stroke neuroplasticity. Astrocytic NF-κB activation is recognized as a key contributor to neuroinflammation; trans-genetic inhibition of astrocytic NF-κB reduced the amount of neuroinflammation and lowered the CSPG (chondroitin sulfate proteoglycan) presence, which may further lead to increased axonal growth (82). Astrocytic MAPK signaling provides another link between neuroinflammation and astrogliosis and thereby may influence axon sprouting after stroke. After an ischemic injury, inflammatory cytokines of the IL-6 family bind to gp130 and activate the downstream MAPK and STAT3 pathways to influence astrogliosis (63). Astrocytic MAPK signaling then mediates the inhibition of GFAP (glial fibrillary acidic protein) overexpression and the release of neurite outgrowth promoting HMGB1 (high-mobility group box 1) from activated astrocytes (83, 84). Astrocytic STAT3 is also a key signaling pathway activated by neuroinflammation, which has been demonstrated to be involved in neuro-regeneration. In the initial stage after injury, neuroinflammation triggers the activation of TLR4/NF-κB/STAT3 signaling, leading to increased expression GAP43 and axonal plasticity (85). Activation of STAT3 also greatly improved axonal outgrowth in CNS neurons possibly through the downregulation of RhoA (86). Although the studies mentioned above indicate that STAT3 activation promotes axonal growth, STAT3 may play a controversial role in axon growth due to its regulation of glia scar formation (87). There are some studies demonstrating the detrimental effects of STAT3. It was reported that inhibition of JAK2-STAT3-involved inflammatory and apoptosis signaling in astrocytes could exert a neuroprotective effect in animals subjected to stroke (88). Recent studies show that regulatory T cells serve as effective inhibitors of STAT3-mediated neurotoxic inflammatory response from reactive astrocytes, which are associated with neuroprotection and improved functional recovery (20, 89). So, amplifying beneficial regulatory T cell responses through IL-2, IL-33, CCL1, or CCL20 may offer new opportunities for neuro-restoration (20).

In addition to responding to cytokines, astrocytes are also an important source of cytokines and chemokines following a neural injury. Cytokines and chemokines from astrocytes are important regulators of neuronal regeneration. CCL2 released from activated astrocytes can attract the infiltration of peripheral monocytes and macrophages (90). In turn, the infiltrated monocytes can regulate the proliferation of astrocytes and the formation of GFAP^+^ glia scar (91). During the subacute phase of stroke, two anti-inflammatory cytokines, IL-10 and TGF-β, from astrocytes can reduce the number of activated microglia, macrophage, and monocytes, which may be related to improved axonal repair (92). Astrocytes are also an important source of pro-inflammatory cytokines. Astrocyte-derived IL-15 aggravated cerebral ischemic injury through their activation of neurotoxic CD8+ T and natural killer (NK) cells (93), neutralizing IL-15-attenuated brain injury and promoting recovery in stroke mice (94). The release of RGMa (Repulsive Guidance Molecule BMP Co-Receptor A) from astrocytes show neurite outgrowth inhibitory effects and also exert immunoregulatory effects by serving as the mediator of Th17 cell-induced neurodegeneration (95). However, at the chronic stage, another inflammatory cytokine, IL-17A, from astrocytes shows different functions compared with T cell-derived IL-17A in the acute phase; astrocytic IL-17A is essential for precursor cell survival and differentiation by the activation of MAPK signaling (96). Furthermore, ischemic brain injury also stimulates the release of neurotrophic factors from activated astrocytes, which may have potential regulatory effects on both neural repair and neuroinflammation (64, 97). As a result, enhancing the anti-inflammatory effects of astrocyte-derived neurotrophic factors may promote the resolution of neuroinflammation. Galectin-1 has been demonstrated to promote astrocyte-derived BDNF secretion and functional recovery in a rat stroke model (98); evidence from the LPS-induced neuro-inflammation model suggest that the potential mechanism might be attributed to the immunoregulatory function of BDNF on microglia responses (99, 100). Given their close relationships with both neuroinflammation and neuronal regeneration, astrocytic immune responses can be manipulated to provide new therapeutic targets for neural damage caused by stroke.

### Alterations in Brain-Resident Immune Cells Through Immune-Related Pathways

PPAR-γ (peroxisome proliferator-activated receptor γ) is a master gatekeeper of neural inflammation. By suppressing NF-κB -mediated inflammation, PPARγ ameliorates the ischemic injury and attempts to prevent neural cell death (101). PPARγ selectively modulates microglia/macrophages to adopt the anti-inflammatory and phagocytic M2 phenotype following cerebral ischemia, facilitating neural regeneration through promoting debris clearance (37). Anti-inflammatory cytokine can further accentuate the PPARγ-dependent phagocytic process being performed by M2 microglia in the ischemic brain (102). PPARγ also indirectly promotes the transition of microglia to the M2 type by suppressing the release of inflammatory cytokines (103). Activating PPARγ signaling through Bexarotene, a retinoid X receptor agonist, has been proved to reduce neuroinflammation and promote debris clearance by M2 polarized microglia (104, 105). However, the potential efficacy of bexarotene in resolving stroke-induced inflammation remains to be explored by future studies.

Toll-like receptors are present on both immune cells and neural cells including microglia, astrocytes, and oligodendrocytes; they provide a link between the immune and central nervous systems. The neuroinflammatory response after cerebral ischemia activates the microglia-mediated TLR signaling, and this subsequently increases the release of inflammatory cytokines and exacerbates neural damage in the acute phase of stroke (106). The negative effect of TLR signaling in neuro-regeneration has been shown in some existing studies. TLR2-deficient mice had higher levels of GAP43 expression, which may allow increased axonal growth following ischemia (107). TLR signaling also limits axon sprouting through the production of CSPG (108). However, there are evidence that have revealed the beneficial role of TLR signaling in stroke recovery. TLR4-dependent clearance of axon debris by microglia has been claimed to be essential for axonal growth; this was demonstrated after either pharmacological or genetic inhibition of TLR4 (109). After clearing axon debris, TLR4 signaling mediates the migration of neuroblasts and the generation of newborn cortical neurons, possibly through producing neurogenic mediators (110). TAM (Tyro3, Axl, and Mertk) receptors, which are upstream regulators of TLR signaling, can affect neurogenesis through inhibiting detrimental MAP kinase and NF-κB activations as well as inhibiting the production of pro-inflammatory cytokines by microglia (111). TLR activation also mediates the myelin sheath formation process of newly regenerated axons. After binding with endogenous ligand high-mobility group box 1 (HMGB1), TLR2 promotes the maturation and survival of oligodendrocytes (112). As a result, future studies are required to find out new strategies that can attenuate the detrimental inflammatory effects of TLR signaling without inhibiting the favorable effect of TLR signaling in neural repair.

### Metabolites of Gut Microbiota Can Alter Brain-Resident Immune Cells

The receptor of SCFA has been demonstrated to exist on the surface of microglia, which means that SCFA can influence the function of microglia. Through SCFA receptor signaling and HDAC (histone deacetylase) inhibition, SCFAs can modulate immune homeostasis in gut, peripheral immune system, and brain (50). Supplement of SCFAs in drinking water ameliorates microglia dysfunction caused by reduced complexity or depletion of gut microbiota (113). In ischemic conditions, SCFAs promote the phenotype transition of microglia and exert neurogenesis and neurite outgrowth permissive effects by activating BDNF–TrkB signaling (114, 115). HDAC inhibition also protects white matter by polarizing microglia/macrophage into the protective type through the GSK3β/PTEN/Akt axis (114). A main component of SCFA-sodium butyrate through its HDAC-inhibiting ability can promote the expression of anti-inflammatory (IL-10) genes and the downstream IL-10/STAT3 pathway in microglia, which can positively promote neurogenesis and axonal growth after a stroke insult (116). In addition to SCFA, metabolites of tryptophan also control the activation of astrocytes and microglia and modulate neuroinflammation through AHR (aryl hydrocarbon receptor) signaling (117). Activation of AHR signaling has been shown to exert an inhibitory effect on adult hippocampus neurogenesis (118). In ischemic brain, upregulated AHR protein level mediates acute neural damage and activates pro-inflammatory gliosis, both of which attenuate neuronal regeneration in subacute or chronic stage (119, 120). Altogether, these findings suggest that functions and phenotypes of brain-resident immune cells can be directly shaped by metabolites from gut microbiota, which can further influence neural regeneration after stroke.

## Therapeutic Strategies Targeting Brain–Peripheral Immune Crosstalk

As discussed in the above sections, crosstalk between brain-resident and peripheral immune system presents an increasingly attractive target for developing neural repair strategies. Investigations aim at finding repair-promoting therapies targeting brain–peripheral immunological interactions were mainly conducted in two perspectives: One is downregulating the detrimental pro-inflammatory responses and downstream pro-apoptotic cascades caused by this interaction, which can protect the newly generated and uninjured brain tissue. The other is upregulating levels of anti-inflammatory factors to facilitate debris clearance, growth factor release, and finally the activation of neuroplasticity-related pathways by cooperation of infiltrated and brain-resident immune cells. Preliminary studies have suggested that pharmacological strategies targeting this crosstalk can create a favorable immune microenvironment and finally lead to better clinical outcomes (Table 1).

**Table 1:** Therapeutic interventions targeting brain–peripheral immune communications in clinical studies.

**Intervention**	**Mechanism**	**Study design**	**Results**	**References**
Fingolimod	Inhibit inflammatory	Open-label, evaluator-blinded, parallel-group	Oral fingolimod was safe within 72 h of stroke onset;	(121)
	T-lymphocyte infiltration	Clinical pilot trial	Oral fingolimod reduced secondary tissue injury and microvascular permeability, attenuated neurological deficits, and promoted recovery	
		Randomized, open-label, evaluator-blind, multicenter pilot trial	Combination therapy of fingolimod and alteplase reduced reperfusion injury, improved clinical outcomes, and was tolerated in acute ischemic stroke patients	(122)
Etanercept	Reduce TNF secretion	Phase I/II parallel double-blind randomized controlled clinical trial	Peri-spinal etanercept promoted mobility of paretic arm and alleviated pain in chronic stroke	(123)
Minocycline	Microglia polarization	Systematic review and meta-analysis	Minocycline improved functional recovery in acute stroke patients	(124)
		Exploratory trial	Combining minocycline with tPA lowered plasma matrix metalloproteinase-9 level	(125)
		Single-blind (outcomes assessor) phase I/II controlled clinical trial	Intra-arterial BMNC transplantation between day 5 and 9 after stroke elevated GM-CSF and PDGF-BB levels, lowered MMP-2 level, showed better functional outcome	(126)
		Open-label, single-arm phase I/II study	Surgical transplantation of bone marrow–derived mesenchymal stem cells was safe and related with improved clinical outcome	(127)
BMNCs	Anti-inflammatory, neurotrophic	phase II, multicenter, parallel group, randomized trial with blinded outcome measure	Bone marrow mononuclear stem cells transplanted intravenously after stroke at a median of 18.5 days were safe but showed no beneficial effects for recovery	(128)
		Randomized, double-blind, placebo-controlled, phase 2 trial	Intravenous bone marrow-derived multipotent adult progenitor cells were safe in acute stroke patients but no significant functional improvement was found	(129)
G-CSF	Bone marrow stem cells	Meta-analysis	G-CSF did not improve neurological outcome in stroke patients	(130)
M2 macrophage		Prospective phase I/II nonrandomized open-label clinical study	Intrathecal M2 macrophage therapy was safe and promoted neurological recovery	(131)

Anti-inflammatory agents were proved to be beneficial for neurological recovery in clinical trials. They can directly counteract the neurotoxic inflammatory responses of brain-resident immune cells or alleviate neuroinflammation by shaping peripheral immune components. Fingolimod (FTY720) is now applied in the treatment for multiple sclerosis, and the therapeutic effects can be attributed to the inhibition of inflammatory T-lymphocyte infiltration (132). In experimental stroke models, FTY720 shows neuroprotective functions by modulating microglia M2 polarization and also improves synaptic plasticity (133, 134). In acute ischemic stroke patients, oral FTY720 was tolerant and exhibited better neural functional recovery and reduced lesion volumes and lower levels of inflammation (121, 122). Etanercept, a selective tumor necrosis factor (TNF) inhibitor, is also a promising strategy targeting neurotoxic immune response. In a Phase I/II clinical trial, peri-spinal administration of etanercept significantly improved mobility function in chronic stroke patients (123). In addition to inhibiting inflammatory processes, boosting beneficial anti-inflammatory processes also shows a positive role in recovery. A bacteriostatic antibiotic minocycline is responsible for aiding alternative M2 polarization of microglia and inhibiting M1 polarization of microglia (135). Oral and intravenous minocycline therapy in stroke patients contributed to functional recovery (124). If applied and combined with rt-PA therapy, minocycline can reduce inflammatory responses triggered by rt-PA (125).

Bone marrow mononuclear cells (BMNCs) are composed of heterogeneous populations of hematopoietic lineage cells, stem cells, progenitor cells, as well as mesenchymal stromal cells. BMNCs promote neural stem cell proliferation and exert regulatory effects in immunological interactions between infiltrated and brain-resident cells (136, 137). Therapeutic efficacy of BMNC transplantation has been tested in several clinical studies. Among all completed trials, bone marrow stem cell infusion was demonstrated to be safe for ischemic stroke patients; intra-arterial and intracerebral delivery were related to improved clinical functional outcomes (126, 127). Furthermore, intra-arterial transplantation in the sub-acute stage also elevated serum levels of several growth factors and lowered levels of inflammatory cytokines (126). Transplantations through intravenous routes were safe but not related to clinical recovery (128, 129), so we speculate that different routes of transplantation may have possible impacts on therapeutic effects. In addition to direct transplantation of BMNCs, granulocyte colony-stimulating factor (G-CSF) can also mobilize BMNCs to ischemic brain and show immunoregulatory effects (138). Although G-CSF treatment promoted alternative microglia polarization, neurotrophic factor production, and axonal sprouting in preclinical studies (139, 140), existing evidence are not sufficient for clinical translation (130), and larger-scale trails are warranted to test the efficacy and to find out the appropriate dose and delivery time course of G-CSF. Furthermore, direct transplantation of autologous repair-promoting M2 macrophage was also proven feasible and led to better neurological recovery in patients (131).

The emerging role of the gut–immune–brain axis in stroke recovery has been investigated in several preclinical studies by fecal microbiota transplantation or metabolite supplement. As a result, targeting gut microbiota provides a new perspective for stroke therapy and needs to be clarified by clinical studies. Existing evidence from completed studies shows that compared with healthy control, stroke patients showed a reduction in SCFA production-related gut microbiota (7); dysbiosis index of gut microbiota was a predictor of unfavorable clinical outcome (141). Furthermore, ongoing clinical studies may also provide further evidence on how the dysbiosis of gut microbiota influences peripheral immune response and stroke outcome, as well as the potential efficacy of dietary supplements (Table 2).

**Table 2:** Ongoing clinical studies about gut microbiota and stroke.

**Status**	**ClinicalTrials.gov Identifier**	**Title**	**Objective**
Recruiting	NCT03470506	A Study of the Relationship of Gut Microbial Composition and Stroke Outcome	Investigate the relationship between gut microbiota, inflammation, and the injured brain
Recruiting	NCT04315922	Multiomics Targeting Microbiome Associated Changes in Stroke Patients	Characterize features of gut dysbiosis in acute phase after stroke and during 3 months follow-up
			Identification of dysregulated microbiome metabolites and immune cells during the 3-month follow-up period
Recruiting	NCT03934021	Gut Microbiota in Acute Stroke Patients	Find out the characteristics of gut-microbiota changes in acute stroke
Recruiting	NCT03812445	Cognition and Gut Microbiome Associated Study of Shanghai People With Acute Ischemic Stroke	Investigate the efficacy of probiotics on altering gut microbiota in ischemic stroke patients
Completed	NCT02008604	Influence of Stroke on the Composition of Intestinal Microbiota	Characterize composition of intestinal microbiome; evaluate relationships between alterations in gut microbiota and immunological parameters (HLA-DR)

As discussed above, therapies targeting brain–peripheral immune crosstalk can be translated into better functional recovery in stroke patients, but more convincing evidence from clinical trials of the later phase are also required for therapeutic translation. Furthermore, functional recovery was mainly assessed by scale score (for example, NIHSS score and mRS score) in existing studies; whether the improved neurological outcomes were gained by functional compensation or neural regeneration still requires verification through *in vivo* imaging methods. The immunoregulatory functions of these therapies were initially demonstrated by markers showing alterations in peripheral immune responses. Recent studies show that the activation state of brain-resident and infiltrated immune cells can also be visualized by medical imaging techniques (142, 143), so more direct evidence of alteration in neuroinflammation can be obtained from stroke patients in future studies. Besides, as the important therapeutic role of thrombolysis in acute ischemic stroke, the safety and potential efficacy of combining these immunomodulatory agents with rt-PA therapy can be explored in future studies.

## Conclusions and Future Directions

Neural repair processes including neurogenesis and axonal growth provide an important anatomical structure for functional reorganization and promoting recovery after an ischemic stroke. There is convincing evidence to suggest that the immune responses induced by ischemic injury can shape the microenvironment and alter the positive and negative regulators of neuronal repair. Immune cells, the cytokines and chemokines that they secrete, and immune-related ligands not only may directly regulate neuronal regeneration but also may act indirectly via their interactions with brain-resident immune cells. As a result, therapies targeting the immune responses after stroke seem to be feasible. However, in the contemporary stage, we can only establish an associative rather than causal link between immune responses and post-stroke neuronal repair, and further mechanistic researches are required to gain a comprehensive understanding of this neuro-immune crosstalk. As brain-infiltrated immune cells did not simply originate from passive diffusion of immune cells in peripheral circulation, one important direction for future studies is to find out upstream regulators of immune cell infiltration and their repair-associated phenotype transition. However, immune responses triggered by these infiltrated cells cannot be simply regarded as regenerative or destructive ones. It is important to boost proper repair-promoting immune responses at a proper time course during recovery. For example, depletion of monocytes at an early stage can reduce neural damage and promote neurogenesis after stroke, but this abolishment of monocytes hinders long-term functional recovery after stroke. Monocyte-derived macrophages share many similar properties with CNS-resident microglia, which poses a challenge to explore their respective roles during stroke recovery. Hence, studies about distinguishing monocyte-derived macrophages from brain-resident immune cells through high-throughput sequencing like single-cell RNA-Seq or through transgenic models deserve to be carried out in the future.

Preliminary studies suggest that gut microbiota and metabolites influence stroke recovery through immunological mechanisms. Gut microbiota and metabolites can directly regulate immune cells from gut, which can then migrate to the ischemic brain and participate in neural damage and repair processes. They also play a pivotal role in regulating permeability of gut barrier and further influence immune responses triggered by translocated microbiota or metabolites in peripheral circulation. Furthermore, they also possess the ability to alter the responses of glia cells in the central nervous system, potentially endowing these cells with neuronal repair regulating functions. However, the ways that these changes in microbiota composition after a stroke influence CNS neurogenesis and axonal growth need to be further clarified and verified through germ-free or fecal microbiota transplantation mouse models in future studies. The efficacy of fecal microbiota transplantation and supplement of probiotics or dietary fiber to stroke patients also need to be tested in clinical practice. Collectively, one can speculate that these communications between peripheral and the brain-resident immune system may represent a new therapeutic approach for reducing the neural damage and promoting the neuronal repair that occurs after a stroke.

## Author Contributions

FL has drafted the manuscript. XC, SZ, and CL have provided editing and writing assistance. JJ, XZ, and ZL have revised it critically for important intellectual content. CZ has approved the final version to be published. All authors have made substantial contributions to this manuscript, contributed to the writing of the manuscript, and read and approved the final manuscript.

## Conflict of Interest

The authors declare that the research was conducted in the absence of any commercial or financial relationships that could be construed as a potential conflict of interest.
